# The synchronous improvement of strength and plasticity (SISP) in new Ni-Co based disc superalloys by controling stacking fault energy

**DOI:** 10.1038/s41598-017-07884-4

**Published:** 2017-08-14

**Authors:** H. Xu, Z. J. Zhang, P. Zhang, C. Y. Cui, T. Jin, Z. F. Zhang

**Affiliations:** 1 Shenyang National Laboratory for Materials Science, Institute of Metal Research, Chinese Academy of Sciences, 72 Wenhua Road, 110016 Shenyang, PR China; 2 University of Chinese Academy of Sciences, 19A Yuquan Road, 100049 Beijing, PR China; 3 Superalloy Division, Institute of Metal Research, Chinese Academy of Sciences, 72 Wenhua Road, 110016 Shenyang, PR China

## Abstract

It is a great challenge to improve the strength of disc superalloys without great loss of plasticity together since the microstructures benefiting the strength always do not avail the plasticity. Interestingly, this study shows that the trade-off relationship between strength and plasticity can be broken through decreasing stacking fault energy (SFE) in newly developed Ni-Co based disc superalloys. Axial tensile tests in the temperature range of 25 to 725 °C were carried out in these alloys with Co content ranging from 5% to 23% (wt.%). It is found that the ultimate tensile strength (UTS) and uniform elongation (UE) are improved synchronously when microtwinning is activated by decreasing the SFE at 650 and 725 °C. In contrast, only UTS is improved when stacking fault (SF) dominates the plastic deformation at 25 and 400 °C. These results may be helpful for designing advanced disc superalloys with relatively excellent strength and plasticity simultaneously.

## Introduction

Ni based superalloys are broadly used as the hot end components in aircraft engines and land-based gas engines for their optimum matching of mechanical properties, such as excellent high-temperature strength, oxidation resistance, creep resistance, and fatigue properties^1, 2^. Since the development of aviation industry and power generation needs higher thermal efficiency and lower emission of CO_2_ and NO_x_, it is an urgent task to elevate the service temperature and improve the high temperature mechanical properties of the Ni-base superalloys. In the last decades, much attention has been paid to develop advanced superalloys with better strength and plasticity following the strengthening mechanisms such as solution strengthening, work hardening, grain refinement and precipitation strengthening^3^. These mechanisms are all based on the blocking effects of the defects against dislocation motion, which ranges from zero-dimensional solute atom to three-dimensional precipitated phase^4^. However, some inevitable shortcomings are also introduced by these strengthening mechanisms, such as element segregation, rapid dislocation recovery and grain boundary oxidization, which will counteract the strengthening effect and diminish the plasticity^1, 5, 6^. Though increasing γ′ content and decreasing γ′ size can improve the strength in superalloy, the plasticity and hot workability will deteriorate consequently when the γ′ content exceeds 50%^7^. Considering the limits of these conventional approaches, developing an alternative way to enhance the strength without the loss of ductility is becoming extremely important.

A novel strengthening mechanism has been discovered in the ultrafine-grained Cu that nanoscale growth twins can induce remarkable strengthening and toughening effects by acting both as dislocation blockers and dislocation slip planes^8, 9^. Subsequently, it is confirmed that nanoscale deformation twins (DTs) can also be introduced to strengthen metallic materials either by deformation at extremely high strain rate at low temperature^10, 11^, or by deformation at moderate level of strain with low-SFE^12^ when dislocation activities are effectively suppressed. Though the severely deformed metals and alloys can yield a great promotion in strength, they will inevitably result in an obvious drop in ductility, exhibiting a typical “banana-shaped” strength-ductility trade-off relation due to the limited capacity of strain hardening of the nanograins embedded with nanotwin bundles^13–15^. Better strength-ductility synergy can be obtained by applying annealing treatment on the severely deformed materials, in order to introduce some larger recrystallized grains that producing extra ductility with sacrificing some strength^16^.

The role of SFE in the deformation behaviors of face-centered cubic (fcc) metals has been extensively studied in Cu and Cu alloys with wide variation of SFE by altering Al or Zn content^17–19^. With the decrease of SFE, wavy-slip will be gradually replaced by planar-slip, because a large separation between partial dislocations induced by lowering SFE inhibits their recombination and extension on the cross-slip plane, causing dislocations to organize themselves into planar arrays or planar slip bands^20, 21^. Apart from dislocation substructures, the twinning tendency can also be promoted obviously due to the reduced critical stress of deformation twinning with decreasing SFE^22–25^. Under tensile deformation, wavy slip and dislocation cell are prevailing in pure Cu, while planar slip and deformation twinning will be activated in the alloyed Cu with lower SFE^18, 19^. It has been demonstrated that this kind of DTs induced by quasi-static deformation cannot only improves the strength, but also enhances the plasticity, creating a unique effect of synchronous improvement of strength and plasticity in Cu-Al alloys^26^. The underlying mechanisms responsible for the SISP are well analyzed on the basis of the intrinsic features of DT and its critical role in the plastic deformation^26^. The improved strength is attributed to that DTs can act as obstacles for dislocation motion and help to hinder the dislocation recovery by separation them into smaller regions. The improved plasticity is ascribed to that the deformation twinning can improve deformation homogeneity due to the enhanced strain-hardening ability, and the twin boundaries can serve as the dislocation slip planes to accommodate plastic strain. Up to now, the existence of SISP has been confirmed in some other alloy systems such as Cu-Zn^18^, stainless steels^27^, which shares the commonality that adding an alloying element to decrease SFE, driving the deformation twinning to dominate plastic deformation.

However, it is worth noting that the SISP introduced by deformation twinning is only discovered in the binary alloys or single-phase alloys subjected to deformation at ambient temperature. While for the multiphase-structured superalloy containing complicated chemical compositions, DTs can hardly form during plastic deformation at ambient temperature according to the published results. Instead, a particular mechanism named as microtwinning has been reported frequently that always operates during the deformation at high temperature and low strain rate such as creep, leading to the formation of microtwins (MTs) in superalloys^28–30^. The prominent differences between MT and DT lie in the twin thickness and twinning mechanisms, the MT has smaller thickness with only 4–50 atom layer, and more complicated twinning mechanism that involving atom diffusion and reordering in a thermally-activated process. However, the influence of MTs on the mechanical properties of crept alloys are scarcely mentioned, because only small content of MTs can be produced at the high-SFE materials. Enhancing the twinnability of superalloys via decreasing SFE has been proved to be practicable, substantial deformation MTs can be introduced during deformation at intermediate temperatures (650–800 °C)^31–34^. It is suggested that these MTs could largely improve the tensile strength and creep life of the tested alloys. Actually, it is hard to give an explicit evaluation on the contribution of MTs to mechanical properties, because the change of SFE is always accompanied with the variation of solution strengthening elements, γ′ forming elements and even the grain size. These factors inevitably affect the deformation mechanism and mechanical property, so the improvement in mechanical property cannot be only attributed to MTs without considering the disturbance of other factors.

Consequently, it is necessary to eliminate these factors in order to get better understanding on the imposed influence of SFE both on the microscopic mechanisms and macroscopic properties of superalloys. Considering that, three newly developed Ni-Co based disc superalloys were chosen as experimental alloys, with Co content increased from 5 wt.% to 23 wt.% by substituting Ni without the change of other alloy elements, for the sake of ensuring the SFE being merely related to Co element. The added Co element in Ni-based superalloys will preferentially partial to the γ matrix, others enter into the γ′ phase by substituting Ni atom, forming (Ni,Co)_3_(Al,Ti)-typed γ′ phase. Adding the Co content can effectively increase the content of electron holes, results in the decrease of SFE in Ni-Co based disc superalloys^35^. The SFE values of 5Co, 15Co and 23Co alloys at room temperature were calculated to be 40.1, 33.3 and 24.9 mJ/m^2^, respectively, via measuring the separation width of two partials of the stacking faults in γ matrix^36^. So, the calculated SFE value actually belongs to the γ matrix of tested superalloys, representing their twinnability during deformation. According to the previous result^37^, the value of SFE is temperature-dependent, increasing temperature can lead to the nonmonotonic change of SFE in superalloys. In spite of this, it is reasonable to deduce that the decrease of SFE by adding Co element may not be affected by temperature on account of the strong effect of Co element on SFE. Besides the composition design, these alloys were then treated in the same hot working and mechanical processing to make sure that they have nearly the same phase constitution, similar grain size, and negligible discrepancy of γ′ volume fraction and size.

## Results

### Typical microstructures of three tested alloys

**Figure 1:**
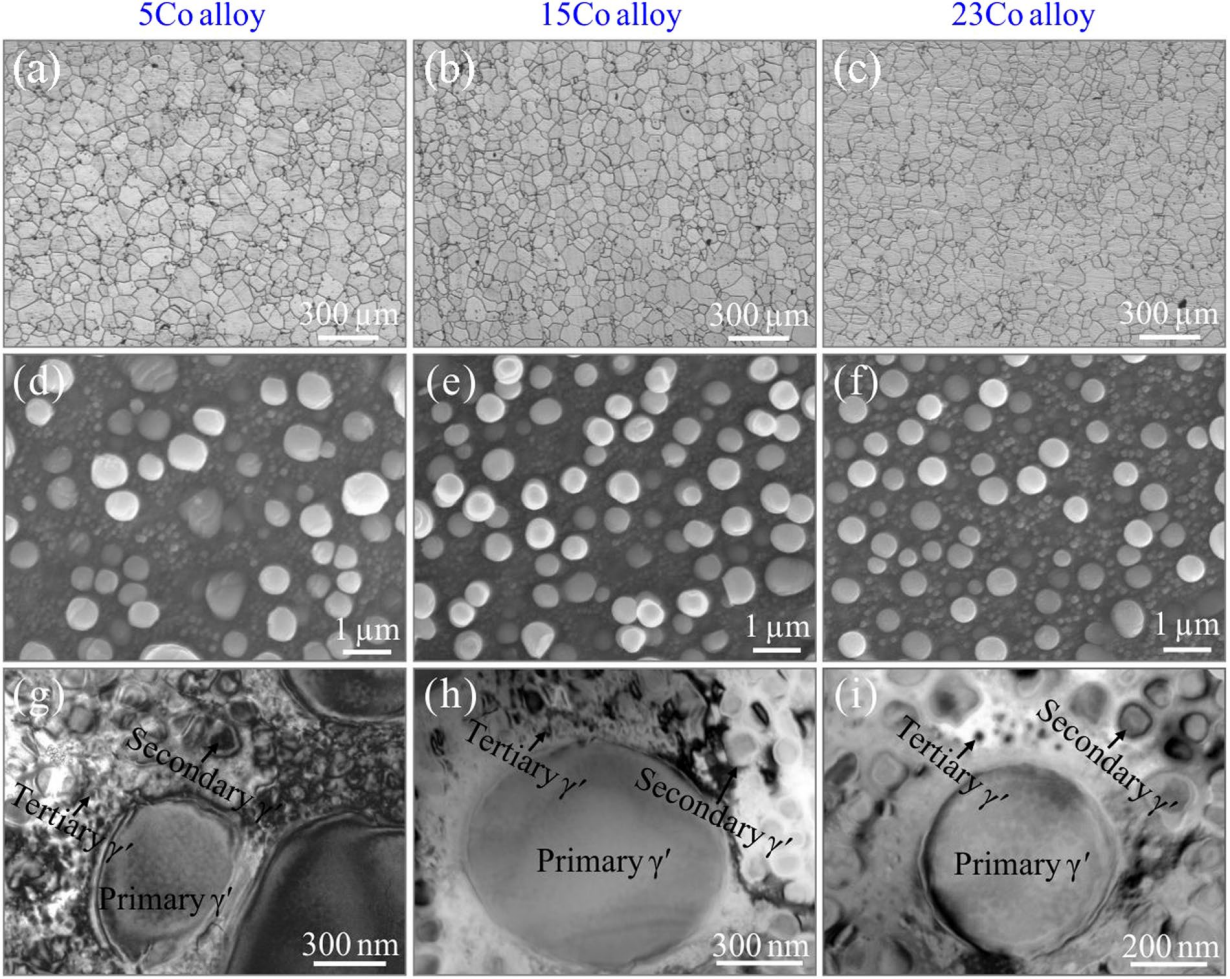
Microstructural observation of three superalloys under different scales. (**a**–**c**) Grain distribution of three alloys by optical microscope; (**d**–**f**) primary and secondary γ′ precipitates in three alloys by scanning electron micorscope; (**g**–**i**) tertiary γ′ precipitates in three alloys by transmission electron microscope.

The original microstructures of the three alloys before tensile deformation are depicted in Fig. 1. The average grain size of three alloys was statistically estimated to be around 35 μm, showing insensitive to the change of Co content, as shown in Fig. 1(a–c). Based on the observations by scanning electron microscope (SEM) (Fig. 1(b–f)) and transmission electron microscope (TEM) (Fig. 1(g–i)), it can be found that dense γ′ precipitates with large variation in size are embedded in the γ matrix, forming hierarchical distributions of precipitates: primary γ′ (>100 nm), secondary γ′ (100–40 nm) and tertiary γ′ (<40 nm). Previous statistical results show that the variation of volume fraction of the congeneric γ′ precipitates is less than 6%, the total volume fraction of γ′ precipitates in the three alloys is about 46.5%^38^. The nearly unchanged volume fraction of γ′ precipitates with increasing Co content can be rationalized that the three superalloys contain the same γ′-forming elements, i.e. Al and Ti. Generally, increasing Co content can enhance the stability of precipitates and lower the γ′ solvus temperature, which widens the processing window and reduces the thermal stresses induced by controlled cooling and quenching in superalloys^31^. It has been reported that high Co addition up to 30% made no difference to the phase constituent, but the size of secondary γ′ will firstly decrease with increasing Co content, and then remain unchanged with further addition of Co above 25%^39^. Since higher Co content will result in lower γ′ solvus temperature in alloys, in this case secondary γ′ will form at lower cooling temperature and have less time to grow up. But in our study, it can be found that Co content has unconspicuous effect on the size of γ′ precipitates due to the same content of Al and Ti elements. Based on the above results, it can be inferred that the three alloys have comparable grain size, strengthening phase constitution and distribution, the discrepancy induced by Co variation can be roughly neglected, which gives us a chance to separately investigate the influence of SFE on mechanical behaviors without the other disturbances.

### The tensile stress-strain behaviors

**Figure 2:**
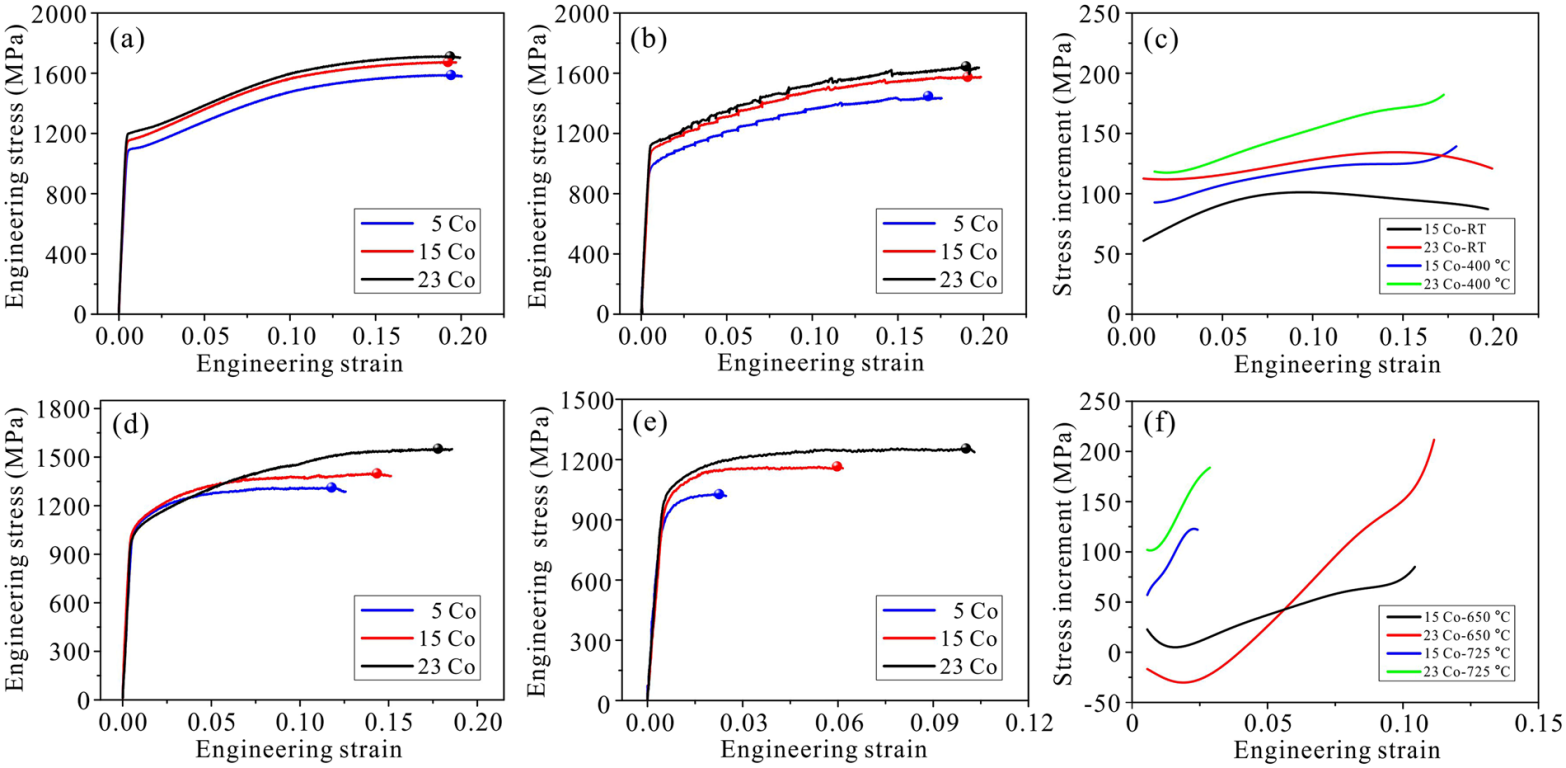
The mechanical behaviors of three alloys under different temperatures. The flow stress-strain curves at (**a**) 25 °C, (**b**) 400 °C, (**d**) 650 °C and (**e**) 725 °C are presented for the three alloys. The colored spheres represent the end of uniform deformation for three alloys, from which ultimate tensile strength (UTS) and uniform elongation (UE) can be obtained. The stress increment is plotted for (**c**) 25 & 400 °C and (**f**) 650 & 725 °C by subtracting the tensile stress of 5Co from that of 15Co or 23Co under similar tensile strain at the same deformation temperature.

The tensile stress-strain curves of three alloys during plastic deformation at temperatures ranging from 25 °C to 725 °C are depicted in Fig. 2. In order to facilitate the comparison of mechanical properties among these alloys, the UTS and UE are labeled by solid spheres with different colors. At 25 °C, the UTS and UE of 5Co alloy reach to 1588 MPa and 19.4%, respectively (Fig. 2a). When the Co content increases to 15% and 23%, the UTS increases by 87 MPa and 124 MPa correspondingly, nevertheless the UE nearly remains the same value. At 400 °C, the UTS and UE of 5Co alloy decrease to 1449 MPa and 16.8%, respectively (Fig. 2b). Compared with the 5Co alloy, the UTS of 15Co and 23Co alloys is 127 MPa and 198 MPa higher, meanwhile the UE of the two alloys is raised by only 2%. Considering the results above, it can be concluded that at relatively low temperatures, increasing the Co content of the present alloys will enhance UTS, but it has no significant effect on UE. In addition, with the increase of Co content, the yield strength of tested alloys will increase obviously. The enhancement in UTS seems to partly derive from the increased yield strength at the initial deformation, during the later deformation, the flow stress of three alloys nearly has comparable variation tendency (Fig. 2a,b). The variation tendency of stress increment against tensile strain between 15Co and 23Co is generally similar at two temperatures, as shown in Fig. 2c, which is plotted by subtracting the tensile stress of 5Co from that of 15Co or 23Co under similar tensile strain at the same deformation temperature. It can be deduced that the three alloys have the similar work-hardening capacity, the discrepancy in UTS among three alloys is mainly resulted from the improvement of yield strength by altering Co content.

When deforming at higher temperature, the three alloys exhibit distinct mechanical behaviors compared with that at 25 and 400 °C. Firstly, the influence of Co content on UTS becomes much more evident, reflecting in larger increasing magnitude of UTS with increasing Co. For instance, the UTS can be increased by 87 MPa and 153 MPa at 650 °C, while it can be increased by 138 MPa and 89 MPa at 725 °C in response to the addition of Co content from 5% to 15% and from 15% to 23%, respectively (Fig. 2d,e). Secondly, the UE of tested alloys can be improved synchronously with UTS as the increase of Co content, in contrast to its insensitive response to Co content under low temperatures. At 650 °C, the uniform elongation of 5Co alloy is 11.8%, and then turns to be 14.3% and 17.8% as the Co content is increased to 15% and 23%, respectively (Fig. 2d). At 725 °C, though the plasticity of tested alloys deteriorates greatly due to the high-temperature oxidation, the UE of three alloys is also enhanced by adding Co content, as shown in Fig. 2e. Thirdly, the yield strength of tested alloys tends to remain unchanged with increasing Co content at two higher temperatures, the improved UTS may be largely ascribed to better work-hardening capacity induced by adding Co. As depicted in Fig. 2f, the stress increment increases with tensile strain at higher rate in contrast with that of low temperatures.

### The SISP tendency of the three alloys

**Figure 3:**
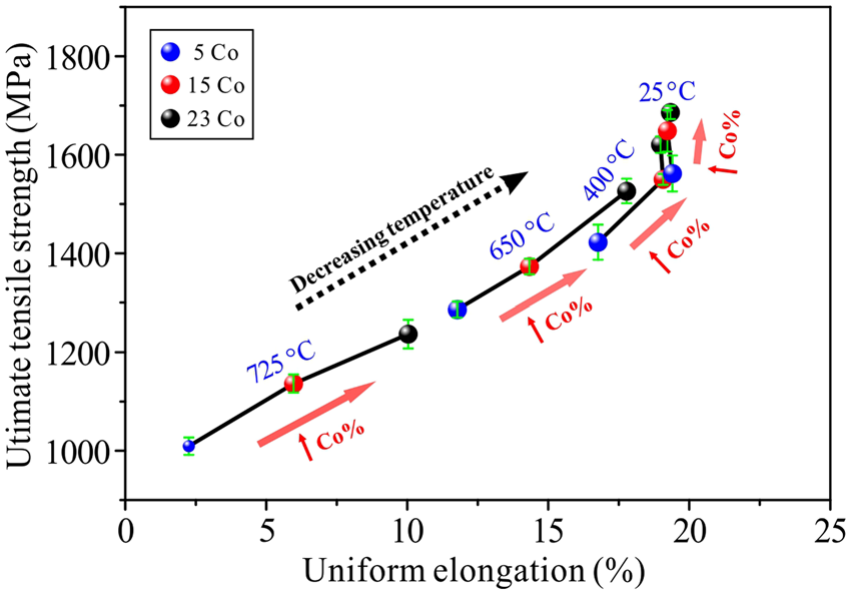
The SISP tendency of three alloys with the decrease of temperature. It shows that adding Co mainly leads to the increase of UTS at 25 and 400 °C, while it can introduce an obvious SISP in the superalloys at 650 and 725 °C.

More intuitive dependence of UTS on UE under different Co content and temperatures are plotted in Fig. 3. It is shown clearly that the SISP tendency of tested alloys is closely related to the testing temperatures, low-temperature deformation shows weak or no SISP, while high-temperature deformation results in obvious SISP. Considering that UTS can be increased by adding Co at the whole temperatures, the key point deciding whether SISP happens or not is the variation trend of UE with increasing Co content. Since the three alloys have similar grain size, strengthening phase constitution and distribution, the distinct performance of plasticity variation with Co element can be ascribed to the microscopic deformation mechanism, which decides the work-hardening rate and capacity of tested alloys. Higher work-hardening rate and better work-hardening capacity will promote uniform deformation, postpone the appearance of necking, and then improve the UE of alloy. As two competing deformation mechanisms, slipping and twinning are found to possess the different work-hardening rate and capacity during deformation. Previous studies in the Cu-Al alloys and Cu-Zn alloys demonstrate that the SISP can be introduced by improving the work-hardening capacity through adding the Al or Zn elements into Cu matrix^18, 26^. It can be deduced that SISP happened in these Ni-Co based alloys is also resulted from the modified microscopic deformation mechanism that being benefit for work hardening. While, unlike the effect of Co content on the tensile properties, the increase of deformation temperature will reduce the strength and elongation of tested superalloys simultaneously, which is different from the tradition FCC metals that the tensile strength will decrease and the elongation will increase with the increase of temperature. This can be attributed to the activation and acceleration of oxidation introduced by increasing the deformation temperature, which will result in the local cracking of grain boundaries and the deterioration of ductility.

### Microstructural evolution under tensile deformation

**Figure 4:**
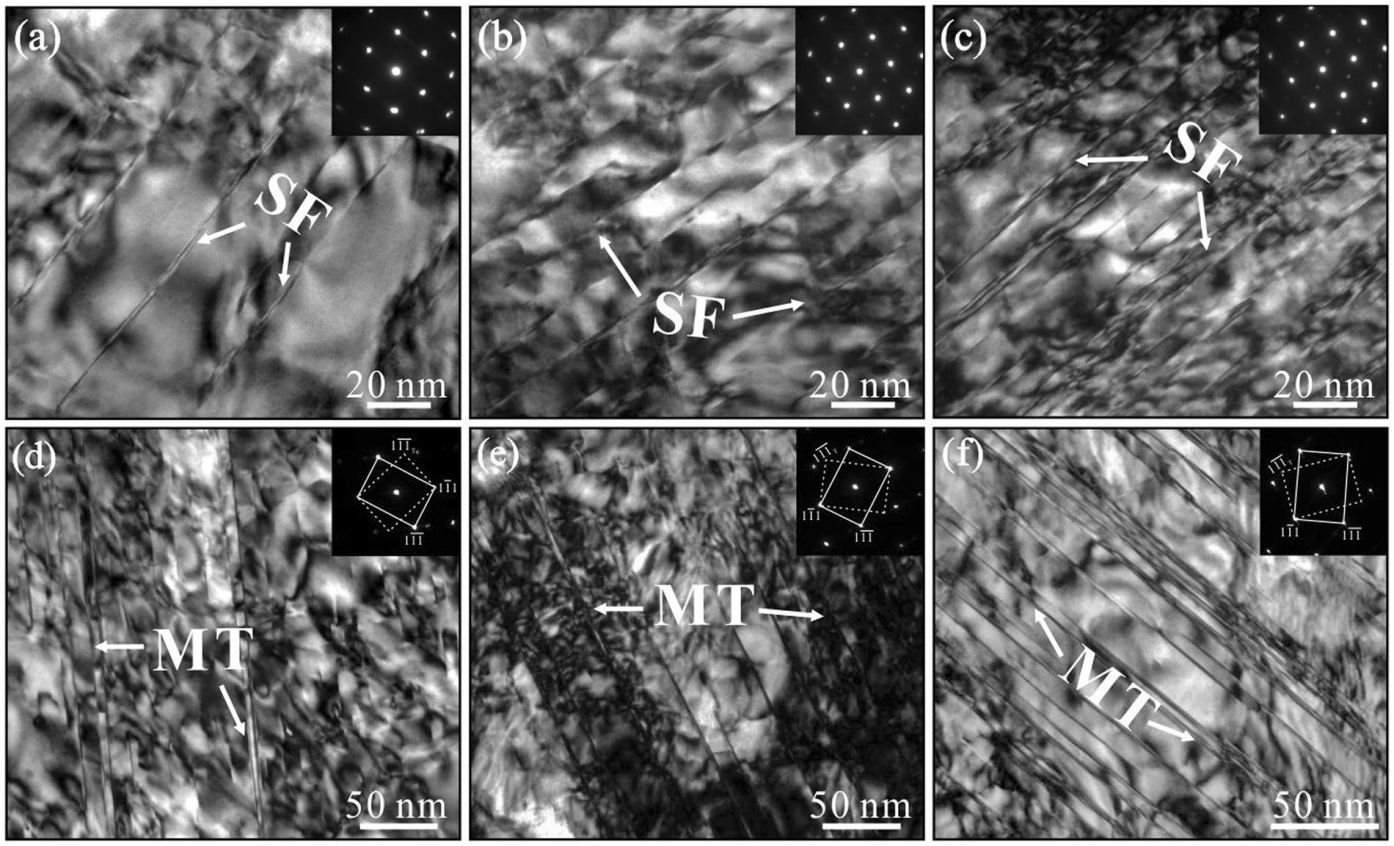
The typical microstructures in three superalloys after deformation at different temperatures. (**a**) 5Co alloy at 25 °C; (**b**) 15Co alloy at 25 °C; (**c**) 23Co alloy at 25 °C; (**d**) 5Co alloy at 725 °C; (**e**) 15Co alloy at 725 °C; (**f**) 23Co alloy at 725 °C, the insets at the top right corner of (**d**), (**e**) and (**f**) are the electron diffraction spots of MTs, SF denotes stacking fault and MT denotes microtwin.

In order to figure out the underlying mechanisms that causing SISP, microstructural observation was carried out to detect the deformation behaviors of the tested alloys under different conditions. It is clearly revealed that there are two distinct mechanisms controlling the deformation at low temperatures and high temperatures respectively, as depicted in Fig. 4. When deforming at low temperatures (25 and 400 °C), typical microstructure is SFs, which present in all tested alloys and shear across the γ matrix and γ′ precipitates continuously (Fig. 4(a–c)). Dislocation structures lying between SFs are displayed as dark irregular stripes due to severe lattice distortion during tensile deformation. MTs can hardly be discovered, SFs accompanied with dense dislocations are the dominant deformation structures for these alloys when deformed at low temperatures. In contrast to that, at high temperature (650 and 725 °C), abundant MT lamellae can be found to shear across the γ matrix and γ′ precipitates in the same way as SFs (Fig. 4(d–f)). Under different deformation conditions, three alloys seem to be deformed by similar deformation mechanisms, the discrepancies introduced by decreasing SFE are not so evident as the previous results. For instance, it has been reported that dislocation slip and/or climb are the main deformation mechanisms in U720Li with SFE of 35.9 mJ/m^2^ (comparable to 5Co alloy) deformed at temperature up to 725 °C, nevertheless dislocation pairs, SFs and MTs will be triggered respectively in the temperature range of 25–650 °C, 650–700 °C, and 700–725 °C for the TMW-4M3 alloy with SFE of 19.9 mJ/m^2^ (comparable to 23Co alloy)^31^. The prominent discrepancies in the influence of SFE on the deformation mechanism between the present three alloys and other disc superalloys may derive from the different alloying systems.

It is shown that the transformation of deformation micromechanisms from SF shearing to deformation microtwinning will occur in the three superalloys in response to the increase of deformation temperature from 400 °C to 650 °C. The improvement in twinning ability with increasing temperature is a unique characteristic in these superalloys, since twins are more prone to form at low temperature or high strain rate in many other metals^40^. The transformation of deformation micromechanisms is not only temperature-dependent, but also related to the relatively low SFE of the three superalloys, which can be rationalized by the corresponding formation mechanisms of the SF and MT. According to the previous results, relatively low SFE is the crucial prerequisite for MT formation in superaloys, regardless of whether they were prepared by casting or forging, or strengthened by solution atoms or γ′ precipitates when deformed at intermediate temperatures^41^. It is evident that two distinct deformation mechanisms are in good agreement the two different mechanical behaviors of three alloys, indicating the different role that played by SF and MT in the strength and plasticity of superalloys. The contribution to mechanical properties made by SF and MT will be discussed in the following section on the basis of their characteristics variation with SFE.

### Influences of SFE on the size and distribution of MT/SF

**Figure 5:**
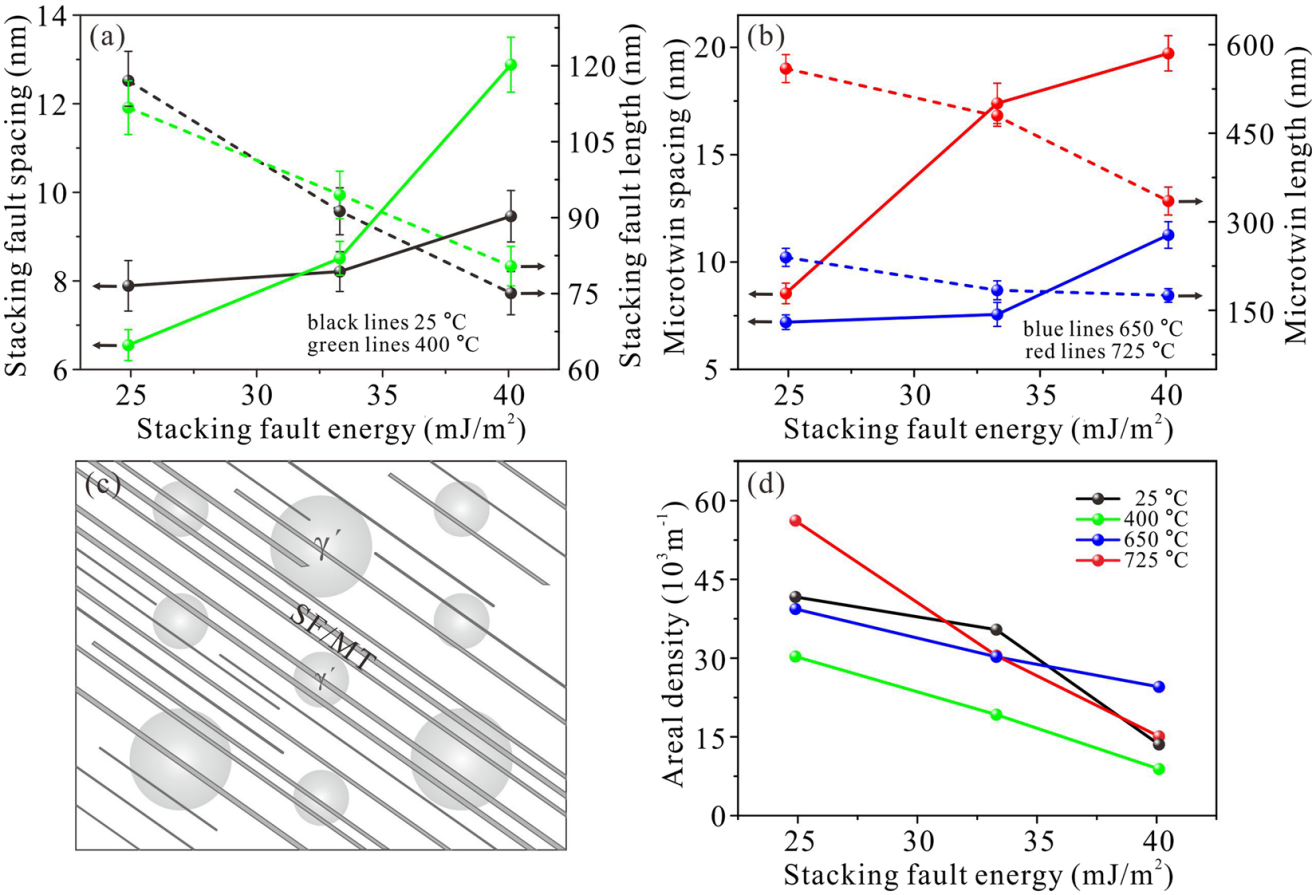
Statistical results on the size and distribution of SF and MT in the three superalloys with different SFE. (**a**) The variation of SF spacing and length with SFE; (**b**) the variation of MT spacing and length with SFE; (**c**) sketch map for calculating the areal density of SF and MT; (**d**) the changes of SF/MT areal density with SFE. 5Co: 40.1 mJ/m^2^; 15Co: 33.3 mJ/m^2^; 23Co: 24.9 mJ/m^2^.

It is widely accepted that SFE plays a significantly role in the nucleation and growth of SFs and MTs. Considering that the thickness of the MT in this study is usually less than 50 atom layers, the change in the thickness with SFE will be neglected, and we only emphasize on the changes in the spacing and length of the SFs and MTs. Statistical calculation was carried out on the variation of SF/MT length, spacing and areal density with SFE in the three alloys, as exhibited in Fig. 5. It shows that the SF spacing of the three alloys decreases and the SF length increases with the decrease of SFE when they were deformed at 25 and 400 °C (Fig. 5a). So, the content of the SFs is increased gradually as the SFE decreases at the two temperatures. Furthermore, the imposed effects of temperature on SF length and spacing vary with SFE. It can be found that the SF spacing and length of 5Co alloy increase obviously with temperature, but those of 23Co alloy decrease as temperature increases. 15Co alloy stays in a transient state that the changes are small with increasing temperature. Similar to SF, the MT spacing of the three alloys also decreases while the MT length increases as the SFE decreases (Fig. 5b). But it is worth mentioning that the influence of SFE on the sizes of MT is much more obvious compared with that of SF. For instance, the MT spacing decreases from 19.7 nm to 8.5 nm and the MT length increases from 335.1 nm to 559.5 nm when SFE decreases from 40.1 mJ/m^2^ to 24.9 mJ/m^2^ at 725 °C. However, the SF spacing just decreases from 12.9 nm to 6.6 nm, and the SF length only increases from 80.4 nm to 111.7 nm under the same SFE range at 400 °C. Besides, the dependence of MT spacing and length on temperature is much more apparent for the three alloys: higher temperatures lead to larger MT spacing and MT length.

The areal density of SF/MT, defined as the total length of SF/MT in a unit area, is carefully measured under different temperatures. Figure 5c is a sketch map of TEM image that contains numbers of SFs or MTs. The total length is obtained by adding up the length of these SFs or MTs from at least 5 pictures, the total area is obtained by adding up the area of each picture, which is calculated by multiplying its length by its width. The areal density of SF/MT is the ratio of total length to total area, the results are depicted in Fig. 5d. It can be inferred that decreasing SFE will lead to the increase of areal density at the whole temperature range, which is in good response to the change of SF/MT spacing and length.

## Discussion

### Twinning mechanism in superalloys

Deformation twinning always prevails in the fcc metals and alloys that deformed at the low temperature and high strain rate while dislocation activities are greatly suppressed. For the coarse-grained fcc metals, several well-known twinning mechanisms have been developed to explain the initiation and propagation of the DTs, such as the pole mechanism^42^, prismatic glide mechanism^43^, and faulted dipole mechanism^44^ according to the characteristics of the dissociation process of the 1/2 <110> dislocation. In the γ′ strengthened Ni-base superalloy, the formation of DTs is always associated with the loading at high temperature and low strain rate^45^. The major differences of twinning procedure between common fcc alloys (e.g. Cu-Al alloys) and superalloys are that the former is single-phase solid solution, easier to be deformed uniformly, while the latter contains two phases of γ/γ′, deformation always starts firstly in the γ corridors, and is trapped at the γ/γ′ interface. Apart from the externally loading conditions, the size, volume fraction and chemical compositions of the γ′ precipitates can strongly affect the activity of trapped dislocations at the interface, which decide the twinning mechanism of superalloys.

Generally, three twinning mechanisms are well-documented for superalloys. The first is zero-strained twinning, it was discovered in a coarse-grained disc superalloys at tensile strain of 3%, this twinning occurs through the cooperative dissociation of three full dislocations or by the overlapping of paired parallel and non-neighboring SFs^46^. The second is SF overlapping introduced twinning, the 1/3 < 1 1 2 > superpartial dislocations directly penetrate into γ′ precipitates, leaving superlattice intrinsic or extrinsic SFs on the parallel and neighboring/non-neighboring {1 1 1} planes. For instance, high-temperature deformation (compression and creep) in some γ/γ′ structured Co-based alloys will result in the dissociation of perfect dislocations into partial dislocations of 1/3 <1 1 2> (leading) and 1/6 <1 1 2> (trailing). Due to the high SFE of L12-Co_3_(Al,W) (~91 mJ/m^2^) as calculated by first-principles^47^, SFs are more frequently produced by the consecutively passage of the leading partials within γ′ phase^48–50^. With lowering the SFE, deformation MTs may also be introduced in the Co-based superalloys when the overlapping tendency of SF is increased. The third is thermally-activated microtwinning proposed by Kolbe^51^, twin formation takes place by a diffusion-mediated reordering within γ′ precipitates after the pairwise passage of identical a/6 <112> Shockley partial dislocation shearing across both γ and γ′ on adjacent {1 1 1} planes.

In this study, deformation at higher temperatures will promote the MT formation, deformation at lower temperatures only produces SFs, this strong temperature-dependent formation of MTs indicates that twinning is likely a thermally-activated process. As is suggested by the third twinning mechanism, when L12-structured γ′ precipitate is sheared by one Shockley partial dislocation, a high-energy complex stacking fault (CSF) will be introduced. The overlapping of these CSFs is probable when a single γ′ precipitate is sheared by one set of Shockley partial dislocations with the same Burgers vector, then pseudo twin will form by the overlapping of at least four neighboring CSFs. These pseudo twins all have an orthorhombic structure with numbers of unstable, high-energy Al-Al nearest neighbor bonds. Then diffusion-mediated atomic reordering need to be provoked between two adjacent CSFs, in order to eliminate the Al-Al bonds, restoring again the initial L12 structure and transforming the pseudo twin to a true MT. This mechanism has been demonstrated by direct TEM observation, theoretical modeling and computer simulation during creep deformation at intermediate temperature^28, 45, 52^. It is shown that reordering as a rate-limiting process requires very simple, vacancy-mediated exchange between Al and Ni atoms, and could be generalized to the formation of superlattice intrinsic and superlattice extrinsic SFs^45^ in superalloys subjected to the similar deformation condition. Based on the above analysis, the reason for the absence of MTs at RT and 400 °C can be rationalized as the high-energy barrier for the atom reordering in the overlapped CSFs, therefore SFs are prevalent in the low temperature range. According to the third theory, if the SFs in γ′ precipitates are formed through the shearing of Shockley partials, they actually are the unstable CSFs. However, it is also possible that SFs may be ascribed to the running through both of γ and γ′ by the 1/3 <1 1 2> superlattice partial dislocations for the high flow stress at low temperatures^53^.

### Controllable deformation mechanism by adjusting SFE

In some fcc binary or single-phase alloys, such as Cu-Al and TWIP steels, the deformation mechanisms are proved to be controllable. Through decreasing the SFE of alloys, the planarity of dislocation slipping is enhanced greatly, the deformation mode will be changed from cross slip to planar slip, and even to deformation twinning^20–25^. With the decrease of SFE, room-temperature SISP can be produced in the Cu-Zn^18^ alloys, Cu-Al alloys^26^ and stainless steels^27^. The deformation mechanisms in superalloys may be greatly affected by the intrinsic factors (e.g. composition, microstructure and SFE) and extrinsic factors (e.g. temperature, strain rate, and loading mode). In superalloys, the deformation micromechanisms are also controlled by its SFE, which may disperse widely on the alloying system and deformation temperature. Generally, the deformation mechanisms of superalloys with medium to high SFE are mainly participated by the dislocation activities. For instance, in the tension deformed René 80 superalloy, γ′ shearing occurs by the slip of APB-coupled 1/2 < 1 1 0 > dislocation pairs on {1 1 1} planes and then cross-slip to {100} planes to act as obstacles to moving dislocations at temperatures below the peak temperature, and then by the viscous slip of two superlattice intrinsic SFs (SISFs)-separated 1/3 < 1 1 2 > superpartials whose cores have split on {1 1 1} planes at temperatures above the peak temperature^54^. Other researchers found that γ′ shearing by dislocation pairs or slip bands dominated the tensile deformation process of traditional polycrystalline superalloys at 600 °C, nevertheless γ′ by-pass was activated and turned to be the main deformation mechanism when the temperature was increased from intermediate (600–850 °C) to high temperatures (beyond 850 °C) regime^55^.

In this study, the changes of temperature and SFE have prominent effects on the deformation behaviors and mechanical properties of three alloys. Firstly, temperature promotes the transformation of deformation mechanisms from SF shearing to deformation twinning. The introduction of SF and MT in the three alloys is also related to the relatively low SFE. Secondary, though the deformation mode of three alloys has no obvious difference at the same temperature, the spacing and length of SF/MT are greatly affected with decreasing SFE. This can be understood in view of the procedure of SF/MT nucleation and propagation. The spacing of the two planar defects depends on the nucleation capability: when the nucleation capability is enhanced, the spacing will be decreased. It has been clarified that the nucleation tendency of SF is mainly decided by the material characteristics and deformation conditions, such as SFE, temperature and strain rate, etc. In this study, under the same deformation conditions, the difference in SF spacing between three alloys could be ascribed to their distinct SFE. As the SFE decreases, two consequences will promote the nucleation of SF: 1) more perfect dislocations will be disassociated following the response to the external force driving; 2) dislocation recovery will be reduced greatly that results in a higher density of Shockley partials in the deformed microstructure. At the MT dominant temperature, as the SFE decreases, more pseudo twin will be generated and the transformation of pseudo twin to real MT will be facilitated. So, the SF/MT spacing will decrease with decreasing the SFE.

Under a stress-free condition, a perfect dislocation in fcc crystalline structure will resolve and form an extended dislocation in order to reduce the energy, which consists of two partials that embedded with an SF ribbon. The spacing of two partials is a balancing of attractive force and expulsive force. The length of extended dislocation is decided by the slipping distance of the leading partial: farther distance results in larger length. It has been demonstrated that the effective force, Orowan stress and SFE have great effects on the slipping of the leading Shockley partial dislocation^56^. Under a shear stress, the decomposition and slipping of dislocations exhibited distinct features from the stress-free condition. Firstly, stress can induce more perfect dislocations to resolve by providing necessary energy for dislocation reaction. Secondly, stress will drive the leading dislocation slipping further since the slip of leading dislocation can assume large portion of strain. In the present study, stress and SFE play a great role in the formation of SF/MT. Alloy with relatively low SFE has lower interfacial energy that may lead to the leading partial slipping further, so longer SF/MT will be generated.

### Strategy for the SISP of disc superalloys

**Figure 6:**
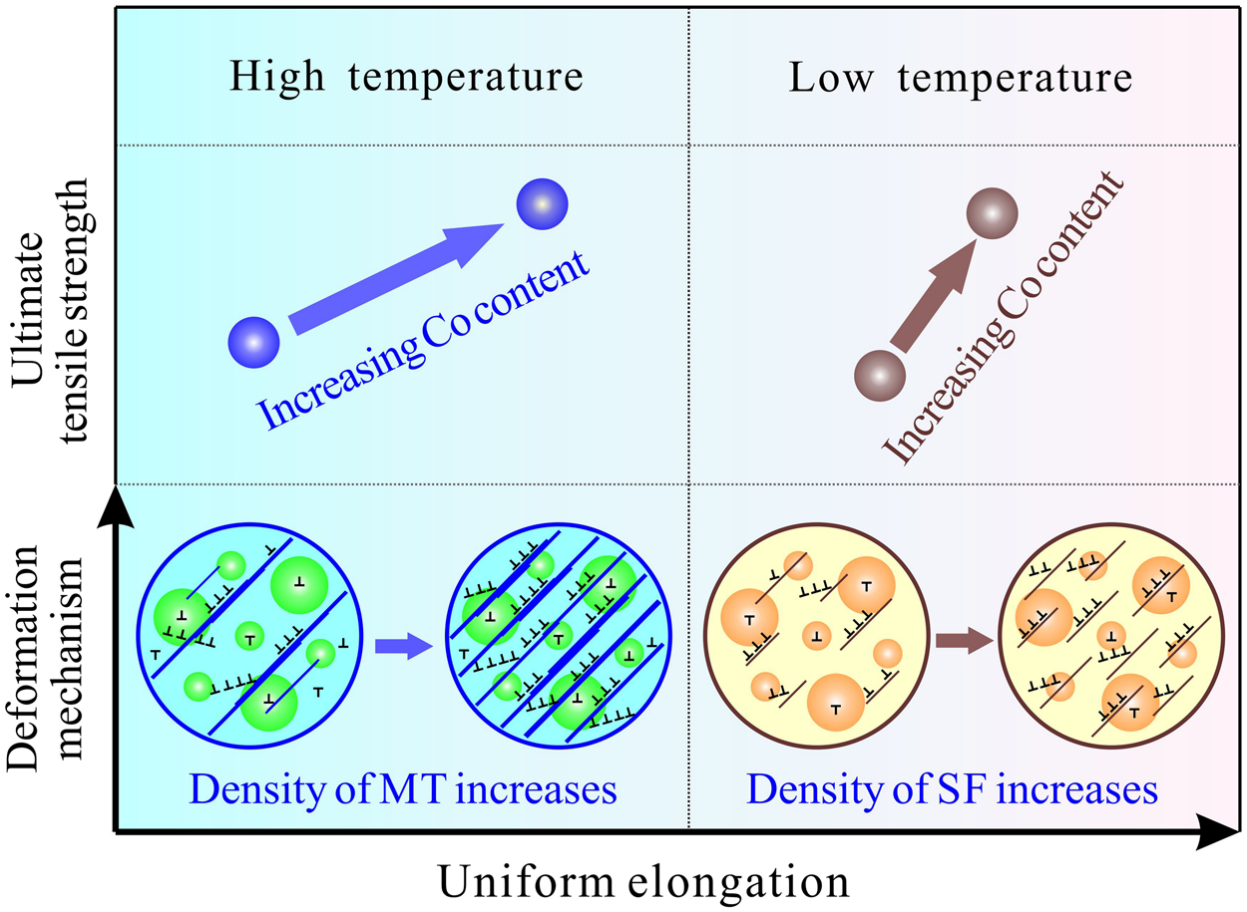
Schematic diagram for describing the relation of microscopic deformation mechanisms and macroscopic mechanical properties.

The above results clearly demonstrate that introducing planar defect of MTs by decreasing SFE can produce SISP for disc superalloys. As summarized in Fig. 6, it is revealed that there are two distinct deformation mechanisms operating during the tensile deformation of the three alloys, SF and MT, dominating at low and high temperatures, respectively. The changes of deformation mechanisms and microstructures with the variation of temperature and SFE will decide the corresponding mechanical properties of the tested alloys. The two deformation mechanisms have different contribution to mechanical properties. At SF dominated temperatures, as the SFE decreases, more SFs can be introduced in the deformation microstructure, which improves the UTS of alloy to some extent due to the inhibition effect of SFs to dislocation slipping as the SFs work in the Cu-Al alloys^57^. However, the UE changes little with the variation of SFE, especially at 25 °C, showing that SFE has weak influence on the UE. This should be ascribed to the limited capacity of storing dislocations in the SF structure with only two or three layers of atoms. In contrast, at the MT operating temperatures, decreasing SFE can increase the density of MTs at a higher amplitude of variation. Previous results based on molecular dynamics simulations showed that a screw lattice dislocation may either propagate into the adjacent twin grain by cutting through the boundary, or dissociate within the boundary plane when confronted with the coherent twin boundary forced by the external stress^58^. While a non-screw dislocation will dissociate into different partial dislocations penetrating into the twin and/or gliding along the twin boundary, producing a sessile dislocation lock at the twin boundary as well^59^. Accordingly, the increased density of MTs will produce more obstacles for dislocation motion, and supply more glide planes to accommodate dislocation motion, resulting in a synchronously improvement of strength and plasticity (SISP) in the Ni-Co based superalloys.

Decreasing SFE is a crucial prerequisite for promoting SISP in disc superalloys, it is successfully achieved by altering Co content in the Ni-Co based disc superalloys. It may also be obtained by adding other single or multiple alloying elements, including W, Mo for solution strengthening, and Al, Ti for precipitation strengthening, and C, B interstitial atoms for GB strengthening, and Cr for improving oxidation resistance, which need further studies in future. According to the author’s knowledge, Co is an alloying element that significantly affects the SFE and nearly does nothing to harm the mechanical properties of tested alloys when its content is below certain value. In addition, since the MTs are generated at the intermediate temperatures, this strengthening mechanism also takes place in the same temperature range, which is just the common service temperatures of the disc superalloys. This finding gives a powerful illustration and attractive prospect of strengthening and toughening nickel-base disc superalloys by mediating SFE.

## Methods

### Sample preparation

The tested alloys were casted as 20-kg ingots by vacuum induction melting following the nominal composition of 5/15/23Co-14Cr-2.8Mo-1.2W-5.6Ti-2.3Al-0.02B-0.02C-0.03Zr-Ni balance (Wt.%). For convenience, the alloys containing 5%, 15% and 23% Co were labelled as 5Co, 15Co and 23Co, respectively. After homogenizing treatment to alleviate composition segregation, these ingots were hot extruded into ϕ35 mm billets with extrusion ratio of 6 at about 1140 °C. The extruded billets were subsequently solution treated at 1100 °C for 4 hours followed by air cooling (A.C), then aged at 650 °C for 24 hours followed by A.C, and finally aged at 760 °C for 16 hours followed by A.C. Dog-bone samples for tensile test were machined into the size of M6 mm × 45 mm with gauge section of M3 mm × 15 mm along longitudinal direction of alloy bars. Before tensile test, all the specimens were electropolished to eliminate the scratches in an electrolyte of 10 pct. perchloric acid and 90 pct. ethanol under 11 V for 1 min at room temperature. The specimens for optical microscopy (OM) observation were etched in a solution of modified Kalling reagent (100 ml HCl, 100 ml methanol and 50 g CuCl_2_). The specimens for SEM were electronically etched in a solution of 80 ml H_2_O +5 ml glacial acetic acid +15 ml nitric acid solution at 1.5 V, for 30 s.

### Mechanical tests

Tensile tests were performed on an Instron 8862 machine at temperatures ranging from ambient temperature to 725 °C with a constant strain rate of 3 × 10^−4^ s^−1^. At least two specimens were tested under each condition to ensure the data reliability. Specimens were kept in electric furnace for 15 min under each setting temperature before test to eliminate temperature fluctuation on the gauge part. Tensile strain was monitored by an extensometer with full range of ±20%. The microscopic mechanisms for tested specimens were investigated using a FEI Tecnai F20 TEM operated at 200 kV. Disks with a thickness of ~500 μm for TEM were cut using electrical discharge machining from the samples perpendicular to the stress axis. The disks were manually ground down to 50 μm and then perforated by a twin-jet electro-polisher in a solution of 10% perchloric acid and 90% ethanol at about 31 V and −22 °C.
